# Process evaluation outcomes from a global child obesity prevention intervention

**DOI:** 10.1186/1471-2458-14-757

**Published:** 2014-07-28

**Authors:** Simone Pettigrew, Jean Michel Borys, Hugues Ruault du Plessis, Lea Walter, Terry T-K Huang, Jeffrey Levi, Jan Vinck

**Affiliations:** School of Psychology and Speech Pathology, CurtinUniversity, Kent St, Bentley, WA 6102 Australia; Secretary General, EIN, 11 rue Galvani, Paris, 75017 France; Coordinator, EIN, 11 rue Galvani, Paris, 75017 France; 984365 Nebraska Medical Center, University of Nebraska Medical Center College of Public Health, Omaha, NE 68198-4365 USA; Milken Institute School of Public Health at the George Washington University, 950 New Hampshire Avenue NW, Washington, DC 20037 USA; Hasselt University, Martelarenlaan 42, Hasselt, 3500 Belgium

**Keywords:** Child obesity, Upstream interventions, Process evaluation, Qualitative research methods

## Abstract

**Background:**

While it is acknowledged that child obesity interventions should cover multiple ecological levels (downstream, midstream and upstream) to maximize their effectiveness, there is a lack of evaluation data to guide the development and implementation of such efforts. To commence addressing this knowledge gap, the present study provides process evaluation data relating to the experiences of groups implementing the EPODE approach to child obesity prevention in various locations around the world. The aim of this exploratory study was to investigate the barriers and facilitators to program implementation in program sites around the world to assist in developing strategies to enhance program outcomes.

**Methods:**

An online survey that included open-ended questions was distributed to the 25 EPODE programs in operation at the time of the survey (May 2012). The survey items asked respondents to comment on those aspects of program implementation that they found challenging and to suggest areas for future improvement. Eighteen programs representing 14 countries responded to the request to participate in the survey, yielding a 72% response rate. The responses were analyzed via the constant comparative method using NVivo qualitative data analysis software.

**Results:**

The main concerns of the various EPODE programs were their ability to secure ongoing funding and their access to evidence-based intervention methods and policy advice relating to relationships with third parties. These issues were in turn impacted by other factors, including (i) access to user-friendly information relating to the range of intervention strategies available and appropriate evaluation measures; (ii) assistance with building and maintaining stakeholder relationships; and (iii) assurance of the quality, independence, and transparency of policies and practices.

**Conclusions:**

The findings are facilitating the ongoing refinement of the EPODE approach. In particular, standardized and tailored information packages are being made available to advise program members of (i) the various evaluation methods and tools at their disposal and (ii) methods of acquiring private partner support. Overall, the study results relating to the types of issues encountered by program members are likely to be useful in guiding the future design and implementation of multi-level initiatives seeking to address other complex and intractable health-related problems.

## Background

The World Health Organization [1, 2] recognizes child obesity as an epidemic that affects both developed and developing nations. Rates of child obesity have increased dramatically in recent decades. It is estimated that there are now more than 30 million overweight children under 5 years of age living in developing nations and a further 10 million in developed nations [3]. These aggregate numbers disguise higher prevalence in developed nations, but rates of increase are higher in developing nations [4].

The prevention and treatment of child obesity is a high health priority because overweight children are more likely than their normal weight counterparts to become overweight adults [1, 5, 6]. As a result, they are more likely to experience a range of health problems across the lifespan, including heart disease, diabetes, stroke, cancer, and mental illness [7, 8]. The increase in prevalence of these diseases at a population level is forecast to have enormous economic consequences due to the very high associated direct and indirect costs [9, 10]. For example, it is estimated that 1-3% of annual health expenditures in most OECD nations are attributable to obesity, with a considerably higher figure estimated for the US (5-10%) reflecting higher levels of obesity [11]. Indirect costs are difficult to quantify, but are expected to exceed direct health costs [12].

Despite the recognized importance of addressing child obesity, little progress has been made at the population level and prevalence levels remain high [1, 13]. Numerous interventions have been implemented in various countries to date including Australia, Belgium, China, Finland, Germany, Israel, Korea, Mexico, and the UK, although the majority of evaluated programs have been conducted in the US (for reviews see [14–16]). The difficulties that have been experienced in addressing child obesity reflect the multifactorial nature of the condition, which has diverse causes including genetics, sedentary lifestyles, the built environment, an increasing amount of food being consumed outside the home, and heavy marketing of unhealthy food products [17–20]. Recent evidence has also pointed to the potential contributions of excessive screen time and sleep deprivation to weight problems in children [21, 22]. Overall, children, along with adults, live in an obesogenic environment that promotes excessive energy intake and discourages physical activity [23, 24].

Given the diverse factors that contribute to obesogenic environments, it is recognized that interventions seeking to address child obesity need to operate at multiple ecological levels [1, 25–28]. The intervention literature categorizes these levels as downstream, midstream, and upstream [29]. Downstream factors are those that influence individual decision-making, such as personal preferences and habits. Upstream factors are those that exist at a community or national level and impact behaviors at a macro level, such as town planning that either encourages or discourages walking for transportation. Midstream factors are the remaining factors that operate at the meso-level, such as individual school policies or family practices that influence children’s participation in sporting activities.

Most obesity programs to date have been downstream in that they have primarily focused on modifying individuals’ decision making processes through education to achieve desirable weight outcomes [30, 31]. Meta-analyses show that downstream obesity interventions typically focus on improving individuals’ diets and/or increasing their physical activity, but that such approaches have had limited success among both adults and children [32–35]. According to these analyses, the short-term nature of such interventions limits their effectiveness and individuals often relapse to their pre-intervention state. In general, downstream interventions reach fewer members of the target population and are less cost effective per capita than interventions targeting midstream or upstream factors [27].

Midstream interventions, typically in the form of family-based interventions, have had some success by altering the home environment. Epstein and colleagues demonstrated significant improvements in child obesity over extended time periods [36, 37]. Their results show the efficacy of focusing on those elements in the child’s family environment that influence food consumption, rather than just focusing on individual decision-making processes. Such an approach reflects the importance of the family environment in influencing children’s weight status [38].

Upstream interventions often involve mandatory or voluntary policies that attempt to shape the environment in ways that make healthy choices easier for individuals [39]. Such interventions reflect the ecological model of behavior that emphasizes the importance of the environment on the actions of individuals [40]. Upstream interventions are increasingly being proposed as more effective means of assisting population-level weight management [23, 28, 31].

Child obesity interventions that cross multiple ecological levels are rare. This situation reflects the high costs, numerous logistical difficulties, and lack of rigorous evaluation data to guide such efforts [41, 42]. It also reflects the focus to date on downstream interventions that rely on educating individuals to improve public health [30]. The few multi-level programs that have been implemented to date have tended to focus on specific areas within individual countries and have featured a strong emphasis on school-based intervention components. Examples include the Shape Up Somerville program implemented in three cities in Massachusetts, USA [43, 44] and the Be Active Eat Well program in a town in Victoria, Australia [45]. A further example that is unique because it is comprehensive, sustained, and international is EPODE. The EPODE approach to child obesity prevention involves addressing a wide variety of factors that impact on children’s health at multiple ecological levels. EPODE originated in France in 2004 in response to burgeoning child obesity rates, with the name representing the phrase "Together Let’s Prevent Childhood Obesity" (Ensemble Prévenons l’Obésité des Enfants’ [46]). More information relating to EPODE is provided further below.

In the context of inadequate evaluation data for comprehensive, multi-level interventions, it has been suggested that the generation of process evaluation data should be an immediate priority to provide vital information for those seeking to implement such interventions [47]. Process evaluations investigate how interventions are implemented, as compared to outcome evaluations that assess the extent to which interventions meet their stated objectives [48]. The present study addresses the need for initial process evaluation data by reporting the experiences of groups implementing the EPODE approach in various locations around the world. The findings will assist in the ongoing refinement of the EPODE approach and are also likely to be useful in guiding the future design and implementation of multi-level initiatives seeking to address other complex and intractable health-related problems (such as programs implemented to address mental health, sexual/reproductive health, youth alcohol consumption, and cardiovascular disease).

The qualitative survey results reported in this article build on an earlier analysis of preliminary EPODE process evaluation data in the form of program-related documents and interviews with implementation staff in three countries [49]. The previous analysis highlighted the importance of garnering political commitment, developing public and private partnerships, engaging in evidence-based social marketing and community-based campaigns, and undertaking rigorous evaluation of program outcomes. The present study extends this work by accessing the experiences and expectations of a much broader range of EPODE-affiliated programs around the world. The rapid expansion of the EPODE approach into numerous countries over multiple continents provides the opportunity to identify the range of issues experienced during the implementation of the program in diverse socio-cultural contexts. A qualitative, exploratory approach was adopted to enable program representatives to nominate and discuss those issues that were most relevant to their particular contexts.

### EPODE

Detailed information relating to the origins and philosophy of the EPODE approach is provided elsewhere [46, 50]. Briefly, EPODE has as its focus the reduction of child obesity through a coordinated, capacity-building approach that involves a broad range of stakeholders and settings. A primary aim is to instil in all children the enjoyment of healthy eating and recreational activities, while avoiding any form of stigmatization. The EPODE approach involves the intentional inclusion of a wide range of stakeholders, ranging from government ministers and national health agencies to community-level organizations, schools, and families. In some contexts, private organizations are also involved. A crucial element in the functioning of the programs is the local project coordinator, who operates as a "go-between" among the national/regional program implementers, the local actors/stakeholders, and the population. This local project coordinator is appointed and remunerated by the local political authorities. Coordinators are active and dedicated members of their communities, and are required to demonstrate knowledge of local political structures as well as a full understanding of the realities of program implementation in the field.

Variations of the EPODE approach have been adopted in numerous countries, each adapting their implementation to accommodate local cultural, social, and political contexts. Adaptation has also occurred within nations, with different programs being implemented in different areas to reflect the varying needs and characteristics of the relevant populations (see http://www.epode-international-network.com/programmes for descriptions of each program). It is estimated that by 2015, variations of the EPODE approach will have been implemented in the form of 40+ large-scale community-based programs involving more than 400,000,000 people.

The EPODE International Network (EIN), a not-for-profit organization, was created April 7, 2011 in Brussels. The objective of EIN is to optimize the effectiveness of the EPODE-based programs around the world by generating global visibility for the EPODE approach, advocating for increased political attention to obesity prevention, encouraging expansion of the scientific evidence base relating to obesity prevention, facilitating information sharing between programs, and fostering links between relevant stakeholders across the public and private sectors [46]. EIN thus provides broad-based support to individual programs to assist them understand and implement the EPODE approach.

## Methods

The study protocol received clearance from the Ethics Committee of the Faculty of Medicine, Hasselt University, Belgium (#CME2013423). An online survey that included open-ended questions was distributed to the 25 EPODE programs in operation around the world at the time of the survey (May 2012). The link to the online survey was distributed via email by the EIN Coordination team. In line with the World Health Organization’s [1] recommendation for child obesity interventions to be effectively monitored and evaluated, the survey was one component of ongoing data collection to obtain information relating to program implementation in various locations and to identify areas where additional support may be required. The survey comprised questions relating to experiences with implementing the EPODE approach, the perceived strengths and weaknesses of the programs as they are delivered on the ground, and any need for additional support. In addition, respondents were invited to make any other further comments they considered relevant. The most senior project coordinator of each program was asked to complete the questionnaire. Respondents could be as brief or as expansive in their replies as they wished.

Of the 25 programs that were members of EIN at the time of the survey, 18 responded to the request to participate, yielding a 72% response rate. These 18 programs represented 14 different countries and four different regions. The Western Europe regional group included France, Belgium, the Netherlands, Portugal, and Greece. The Eastern Europe group encompassed Slovakia, Poland, and Romania (two programs). The South America group included Mexico (three programs), Brazil, and Chile, and the Asia Pacific region was represented by Singapore, Australia (two programs), and New Zealand. The non-responding programs were almost all located in South America, with the exception of one program located in the Asia Pacific region. The survey was produced in English, and all responses were received in English. In the findings presented below, respondents’ quotes are attributed to the regions from which they originate to provide anonymity at the program level.

The survey responses were imported into NVivo 10 (QSR International Pty Ltd, Australia) for coding and analysis. An exploratory approach to analysis was adopted that involved using the constant comparative method to identify commonalities and differences in responses across the various programs to generate a thematic analysis [51]. Data were coded according to location and the various issues raised by respondents (e.g., importance of government funding and optimizing media coverage). Analysis involved a combination of interrogating individual nodes (the locations at which coded data assigned to a particular conceptual category are stored within NVivo) and running matrix searches to facilitate closer inspection of the themes identified in the data. Various trustworthiness techniques were used to enhance the quality of the interpretation, including the use of verbatim quotes (allow direct access to the respondents’ views), purposive sampling (by approaching senior managers with primary responsibility for program implementation), and peer debriefing among the research team [52].

The themes identified in the data related to those aspects of program delivery that were most salient to the respondents. In accordance with the exploratory approach adopted, the issues prioritized by respondents became the focus of the findings reported below.

## Results

The respondents commented on their perceptions of the performance of their own programs and discussed those aspects of program delivery that they felt were especially successful or problematic. They also provided a ‘wish list’ of the additional forms of support they felt could assist them improve outcomes in their particular contexts. Of note was the consistency of responses, with few variations evident by region. The sections below outline these findings, with relevant quotes provided from the survey data. Where appropriate, the quotes have been adapted to account for the second-language status of English in many of the participating countries.

Overall, the respondents found participation in an international network of organizations undertaking similar activities to be useful in supporting their efforts:

*We are very happy to be part of EIN. We value the opportunity to share our experiences and at the same time learn from all the countries that are participating. It is an opportunity for our program to grow and strengthen. (South America)*

Their pride in their association with EIN was reflected in the expectation that the organization will maintain high standards and transparent independence to optimize the short- and long-term success of the network. An important aspect of this independence was perceived to be clear demarcation between industry funding and industry involvement in strategy development and implementation:

*There needs to be an explicit statement about the role (of corporate sponsors) in all communications. (Asia Pacific)*

*(We want EIN to) be completely independent from conflict of interests and be able to show it. (Western Europe)*

Part of the process of maintaining high standards was noted to be an ongoing commitment to publishing results in the scientific literature to provide tangible evidence of the credibility and effectiveness of the comprehensive intervention approach:

*(We want EIN) to publish reports on the results of the individual programs in international scientific fora. (Western Europe)*

*(We want EIN) to hold the program to high scientific standards so that we deliver definitive new evidence and to disseminate the results widely. (Asia Pacific)*

### Main factors influencing effectiveness of delivered programs

Frequent reference was made to the importance of strong relationships with relevant stakeholders to enhance the functioning of the program. Nominated stakeholders included policy makers, community groups, schools, research institutions, the media, and private sector organizations. While not all respondents could report strong affiliations with all these stakeholders, their ability to progress their objectives was attributed to establishing and maintaining effective working relationships with at least one or more such groups. This reflects the philosophy of EIN, which emphasizes the need to work with a range of collaborators across multiple facets of society to provide a broad-based approach to obesity prevention.

*We have initiated strong, sustainable, long-lasting partnerships with schools, summer day-camps, and local organizations…We have excellent relations with the press and media. (Western Europe)*

*We have had a good response from social, private, and academic sectors. (South America)*

*There is strong political commitment and strong support from schools for our obesity prevention programs. (Asia Pacific)*

The primary factors that were mentioned as limiting the success of respondents’ programs to date included inadequate financial support from public and/or private sources and the challenges associated with generating the outcome data required for rigorous evaluation. These weaknesses were also the focus of statements relating to desired forms of additional support, as outlined further below.

*All our efforts to obtain sources for financing program from public sources were unsuccessful, even though all political representatives at national (minister of health, minister of education and sport) and municipal levels were very impressed by the aim of program and its strategy. (Eastern Europe)*

*We feel as though our actions are limited due to our budget. The development and implementation of large actions could be improved with more resources. (Western Europe)*

*We need assistance with the correct application and interpretation of statistics required. (South America)*

*There is a large gap between the socio-economical levels of our participants, which represents a real challenge in terms of conducting an accurate, significant evaluation of the impact of our actions. (Western Europe)*

### Desired forms of additional support

Respondents’ experiences with implementing their programs provided them with insights into those areas of high-level administrative support that are important for optimizing fidelity within and across programs. A particularly critical element was considered to be an extensive database that serves as an information resource for participating groups. The primary function of such a database would be to provide comprehensive information about the activities that have been undertaken by the various participating programs and the results of these activities. Inclusion in the database of best practice examples was considered to be potentially very useful in preventing individual programs from having to learn what works through trial and error. Respondents also suggested that such a database could include a repository of scientific information relating to obesity, its causes, and possible solutions. Ideally, this information would be provided in user-friendly formats that could be used in discussions with policy makers and other key stakeholder groups.

*(The database could be used to) share activities, best practices, results (BMI), best practice to involve new financial partners, and best practice in the evaluation of local mobilization…(There could be) a review of the latest news on obesity and overweight prevention in children and a review of methods that have proved their efficiency on the field (Western Europe)*

Another form of support that was mentioned frequently related to program evaluation measures and procedures. Many respondents expressed a desire for fully specified evaluation criteria and instruments to enable them to produce outcome results that are comparable across programs using methods that would be adequately rigorous for publication in the scientific literature. It was acknowledged that physiological data are especially difficult to access, and assistance in developing appropriate procedures to facilitate the capture of these data was sought.

*(It would be good) to standardize the methods for anthropometric measurements in children – weight and height but also abdominal circumference…And to standardize the food and lifestyle questionnaire according to children’s age and adapted to traditional food consumption and life customs for each country. (Eastern Europe)*

*(We need) help to improve the tools that we are using to evaluate our programs. (South America)*

*We could use more accompaniments in the evaluation process. We need evaluation processes which are meaningful and incontestable. (Western Europe)*

*(We need) ways to evaluate that are cost effective and inform policy in a practical way. (Asia Pacific)*

*(It would be good to have a) common evaluation tool - sustainable methodology for implementation, research, and evaluation. (Asia Pacific)*

Similarly, some respondents were desirous of assistance and guidance in developing the communication strategies that are used to disseminate health messages as part of their programs. The specialist expertise required for effective message development and dissemination was not always readily available in local contexts, and it was felt that EIN could assist with these processes at a central level. This concern was most apparent among the Eastern European respondents:

*(We would like) assistance with the improvement of the education methodology - new ways of education and spreading the information among the program’s participants. (Eastern Europe)*

*(We would like assistance with an) overall communication strategy, for example including national institutions and organizations related to public health, sport, etc. (Eastern Europe)*

A final primary area of desired support related to the negotiation and management of relationships with public and private organizations. This was perceived to be a particularly complex issue because of: (i) the need for ongoing financial support to achieve program sustainability and hence the need to develop financial relationships with third parties; (ii) the differing cultural contexts which prevent a unified approach across countries; and (iii) the varying levels of perceived acceptability of food industry support of child obesity interventions in different countries. This complexity appeared to result in considerable stress for some respondents, especially those located in countries where government funding was less likely to be available.

*We have been totally unsuccessful in obtaining support from the government, municipalities, funds, and private investors. We could operate this project with a minimum 20 people on full time job, but we cannot!!! We would like to meet global representatives of private sector companies who could help us. (Eastern Europe)*

*(We would like EIN) to share best practices in terms of public-private partnership and the methodology behind the success stories. (Eastern Europe)*

*(We would like access to) a list of companies interested in investing and participating with government partnerships. (South America)*

A related issue was the desire for expert assistance to influence food advertising practices. It was understood that food advertising is an important determinant of children’s diets, and hence that comprehensive child obesity interventions need to include strategies to address the current emphasis in food advertising on unhealthy foods. Some respondents reported that they would value assistance in working with the food industry to address this issue.

*(We would like EIN to) build partnerships with the food industry to regulate advertising of their products and encourage involvement in nutrition education strategies for consumers. (South America)*

*(We would like EIN) to provide counselling to stakeholders to improve food marketing and strategies to improve and maintain child general health. (Eastern Europe)*

## Discussion

**Figure 1:**
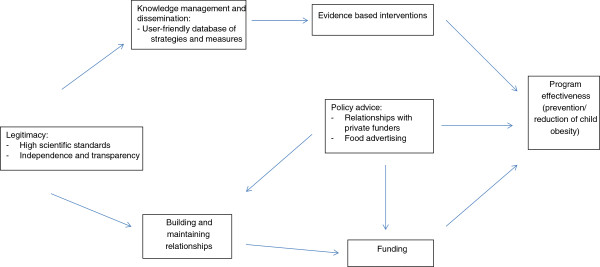
**Identified issues relating to program implementation.**

The qualitative, exploratory approach adopted in this process evaluation study permitted those issues of most importance to respondents to emerge and be integrated into an overall interpretation of the high-level factors affecting program delivery. Ultimately, the primary concern of EIN member programs is their effectiveness in terms of addressing child obesity. As depicted in Figure 1, effectiveness was viewed by the respondents to this survey to be dependent on numerous factors, the most important of which were their ability to secure ongoing funding and their access to evidence-based intervention methods and policy advice relating to relationships with third parties. These issues were in turn impacted by other factors, including (i) access to user-friendly information relating to the range of intervention strategies available and appropriate evaluation measures; (ii) assistance in building and maintaining relationships; and (iii) assurance of the quality, independence, and transparency of EIN policies and practices.

Issues relating to funding are well recognized in the health intervention literature [53, 54], and hence the salience of this concern was unsurprising. However, the differing financial support environments of the various programs and the resulting inability to establish consistent guidelines for procuring and maintaining funding highlight the difficulty of navigating this issue in multi-country collaborations. The project coordinators participating in the study indicated that the financial resourcing of their programs was reliant on a complex interplay of factors that left them vulnerable on several fronts. The outcome was that they sought (i) assistance in identifying and approaching potential contributors, (ii) guidance in establishing appropriate relationships with these entities, and (iii) assurance of the legitimacy, independence, and effectiveness of the EPODE approach that could be conveyed to potential and current funders and other stakeholders.

Similarly, the desire expressed by the study participants for evidence-based strategies reflects best-practice recommendations in the literature [1, 2, 27, 53, 55, 56]. Understanding the information needs of those implementing programs is a vital element of the intervention development process [57]. The project coordinators felt that they would be best equipped to implement evidence-based strategies if they had access to a user-friendly database that listed the different types of interventions that have been used in the child obesity context and the conditions under which they were found to be effective. This database would ideally also include validated evaluation measures that the various programs could use to assess their efforts and generate data that are directly comparable to those being produced by other programs. In this way, they could contribute to the evidence base to inform future intervention implementation in their own program, other EPODE-affiliated programs, and child obesity prevention efforts in general.

On the basis of the study findings, EIN has commenced the introduction of a range of initiatives to enable them to better support implementation activities across the large and growing number of programs. Table 1 lists the range of issues raised by the study participants, the initiatives that have already been implemented in response to these concerns, and initiatives that are in the process of being developed.

**Table 1:** **EIN responses to identified implementation issues**

Area of support	Initiatives in process	Initiatives planned
Evaluation	- Creation of the EIN Scientific Advisory Board (SAB) with multidisciplinary experts.	- Funding and establishing an evaluation framework for 3 EIN member programs.
	- Taskforce on Global Evaluation Project.	- Tailored coaching in evaluation for EIN members.
	- Taskforce on publications.	- Dissemination of the WHO’s appraisal tools to EIN member programs.
	- Facilitating EIN SAB participation in international meetings on program evaluation.	- Development of a common evaluation and intervention taxonomy.
	- Ongoing surveys of evaluation issues among EIN member programs.	
Funding	- Capacity and capability workshops for member program coordinators.	- Leverage funding for evaluation activities.
	- Travel and accommodation expenses covered for specific interventions/meetings.	
	- Monitoring of public and private opportunities for program funding.	
	- Tailored support provided to EIN member programs to identify funding partners and develop relationships with public officials.	
Public-Private partnerships	- Establishment of a Public-Private Partnership (PPP) Taskforce to ensure full transparency and avoid conflict of interest issues.	- Create a common commitment charter for private partners to ensure full transparency and avoid conflict of interests.
	- Ongoing surveys on PPP administered to EIN member programs to identify levers and challenges of partnerships.	
Advocacy and Awareness	- A biannual Global Obesity Forum is held for member programs and other stakeholders.	
	- Regular meetings with politics/scientists/policy-makers and partners.	
	- Dissemination of scientific publications.	
	- Participation in national and international conferences (+30 per year) and collaboration with major obesity conferences (e.g., Pan American Conference on Obesity, American Nutrition Society Conference).	
	- Ongoing communications (website, twitter, YouTube, newsletters, flashnews, infographics, etc.).	
Methodological guidance	- Organisation of "Kick-off meetings" for new programs.	- Specific methodological advice on request.
	- Running capacity building workshops.	- Development of a comprehensive and user-friendly database.
	- Organisation of Regional Obesity meetings (European, Latin American, and Asia-Pacific).	

A limitation of this study was the reliance on data from a single representative from each program. It is possible that those in other roles, especially those involved in day-to-day program implementation, may have different perceptions and needs. Future research could access a broader range of program representatives. A further limitation was the use of an online survey to collect qualitative data. This approach facilitated timely and simultaneous data collection from the various programs, but lacked the ability to probe respondents for greater detail or to seek clarification on the issues raised. An alternative approach for future studies could be the use of interviews – either face-to-face or via online videoconferencing systems (e.g., Skype).

The major strength of the study was the high response rate, with the final sample reflecting project coordinators from a wide range of nations. The findings are therefore likely to be representative of the issues faced by the diverse programs affiliated with the EIN, and the initiatives implemented in response should be well-placed to commence addressing these issues. However, given the global prevalence of child obesity, the recognized substantial adverse health consequences of the condition, and the limited resources of EIN as an NGO, higher-level assistance to address the identified issues appears warranted. For example, a best-practice database relating to child obesity prevention strategies and related evaluation measures may be better managed by an entity such as the World Health Organization that has ready access to the required expertise and technical support.

Another potential area of support at this higher level would be the development of standards for public-private partnerships in health interventions in general and child obesity prevention in particular. The involvement of private organizations in health promotion can invite criticisms relating to the possibility of interference and suboptimal outcomes [58]. Current recommendations relating to child obesity prevention interventions by the World Health Organization [2] note the importance of the private sector as an employer and provider of goods and services, but do not offer specific guidance in managing contractual relationships involving contributions to health interventions. Such guidance could include disclosure requirements and independent monitoring processes.

## Conclusion

Implementing an obesity prevention intervention on a global scale brings many challenges. Previous analyses of national health promotion programs have highlighted the difficulties associated with political commitment and resource constraints [53, 55]. Increasing the scale of an intervention to an international level has the potential for these problems to magnify and multiply. By definition, such an undertaking will lack the auspices of a single government body. In the context of child obesity, the responsibility of implementing an international child obesity intervention has fallen to a non-government organization. EIN has rapidly accumulated numerous community-based initiatives around the world that in combination impact the lives of many millions of children [46]. EIN promotes the EPODE methodology in many culturally diverse countries, encouraging adaption to each specific location rather than promoting a one-size-fits-all approach. The present study provides insight into the process issues experienced by those implementing the intervention on the ground and identifies potential means of addressing these issues to better facilitate implementation in existing and new sites. The findings have the potential to be of broader relevance given the increasing need for multi-level, comprehensive interventions to address non-communicable diseases and their multi-factorial causes. The findings illustrate the importance of ensuring that program staff members have ready access to best practice information about intervention strategies and evaluation measures in user-friendly formats. The provision of advice and assistance relating to establishing and maintaining relationships with funding bodies is also likely to be of particular value.

## References

[1] World Health Organization (2010). Population-based prevention strategies for childhood obesity. Report of a WHO forum and technical meeting, Geneva, 15–17 December 2009.

[2] World Health Organization (2012). Prioritizing areas for action in the field of population-based prevention of childhood obesity.

[3] World Health Organization (2013). WHO (2013) Obesity and overweight, Fact sheet No. 311.

[4] Ng M, Fleming T, Robinson M, Thomson B, Graetz N, Margono C, Mullany EC, Birukov S, Abbafati C, Abera SF, Abraham JP, Abu-Rmeileh ME, Achoki T, AlBuhairan FS, Alemu ZA, Alfonso R, Ali MK, Ali R, Guzman NA, Ammar W, Anwari P, Banerjee A, Barquera S, Basu S, Bennett DA, Bhutta ZQ, Blore J, Cabral N, Nonato IC, Chang J-C (2014). Global, regional, and national prevalence of overweight and obesity in children and adults during 1980–2013: a systematic analysis for the Global Burden of Disease Study 2013. Lancet.

[5] Katan MB (2009). Weight-loss diets for the prevention and treatment of obesity. N Engl J Med.

[6] Serdula MK, Ivery D, Coates RJ, Freedman DS, Williamson DF, Byers T (1993). Do obese children become obese adults? A review of the literature. Prev Med.

[7] James WPT, Jackson-Leach R, Ni Mhurchu C, Kalamara E, Shayeghi M, Rigby NJ, Nishida C, Rodgers A, Ezzati M, Lopez AD, Rodgers A, Murray CJL (2004). Overweight and obesity (high body mass index). Comparative Quantification of Health Risks.

[8] Sanderson K, Patton GC, McKercher C, Dwyer T, Venn AJ (2011). Overweight and obesity in childhood and risk of mental disorder: a 20-year cohort study. Aust N Z J Psychiatry.

[9] Wang YC, McPherson K, Marsh T, Gortmaker SL, Brown M (2011). Health and economic burden of the projected obesity trends in the USA and the UK. Lancet.

[10] Wolfenstetter SB (2012). Future direct and indirect costs of obesity and the influence of gaining weight: results from the MONICA/KORA cohort studies, 1995–2005. Econ Hum Biol.

[11] Organisation for Economic Co-operation and Development (2012). Obesity Update 2012.

[12] Lehnert T, Sonntag D, Konnopka A, Riedel-Heller S, König H-H (2013). Economic costs of overweight and obesity. Best Pract Res Clin Endocrinol Metab.

[13] Birch LL, Ventura AK (2009). Preventing childhood obesity: what works?. Int J Obes.

[14] Ho M, Garnett SP, Baur L, Burrows T, Stewart L, Neve M, Collins C (2012). Effectiveness of lifestyle interventions in child obesity: systematic review with meta-analysis. Pediatrics.

[15] Monasta L, Batty GD, Cattaneo A, Lutje V, Ronfani L, van Lenthe FJ, Brug J (2010). Early-life determinants of overweight and obesity: a review of systematic reviews. Obes Rev.

[16] Han JC, Lawlor DA, Kimm SYS (2010). Childhood obesity. Lancet.

[17] Burns C, Jackson M, Gibbons C, Stoney RM (2002). Foods prepared outside the home: association with selected nutrients and body mass index in adult Australians. Public Health Nutr.

[18] Lichtenstein AH, Appel LJ, Brands M, Carnethon M, Daniels S, Franch HA, Franklin B, Kris-Etherton P, Harris WS, Howard B, Karanja N, Lefevre M, Rudel L, Sacks F, Horn LV, Winston M, Wylie-Rosett J (2006). Diet and lifestyle recommendations revision 2006. A scientific statement from the American Heart Association Nutrition Committee. Circulation.

[19] Morrill A, Chinn C (2004). The obesity epidemic in the United States. J Public Health Policy.

[20] Zimmerman FJ (2011). Using marketing muscle to sell fat: the rise of obesity in the modern economy. Annu Rev Public Health.

[21] Chahal H, Fung C, Kuhle S, Veugelers PJ (2013). Availability and night-time use of electronic entertainment and communication devices are associated with short sleep duration and obesity among Canadian children. Pediatr Obes.

[22] Maher C, Olds TS, Eisenmann JC, Dollman JL (2012). Screen time is more strongly associated than physical activity with overweight and obesity in 9- to 16-year-old Australians. Acta Pædiatrica.

[23] Osei-Assibey G, Dick S, Macdiarmid J, Semple S, Reilly JJ, Ellaway A, Cowie H, McNeill G (2012). The influence of the food environment on overweight and obesity in young children: a systematic review. BMJ Open.

[24] Swinburn B, Egger G (2002). Preventive strategies against weight gain and obesity. Obes Rev.

[25] Huang TT, Drewnowski A, Kumanyika SK, Glass TA (2009). A systems-oriented multilevel framework for addressing obesity in the 21st century. Prev Chronic Dis.

[26] Frerichs L, Perin DMP, Huang TTK (2012). Current Trends in Childhood Obesity Research. Curr Nutr Rep.

[27] Sacks G, Swinburn B, Lawrence M (2009). Obesity Policy Action framework and analysis grids for a comprehensive policy approach to reducing obesity. Obes Rev.

[28] Waters E, Sanigorski AS, Hall BJ, Brown T, Campbell KJ, Gao Y, Armstrong R, Prosser L, Summerbell CD (2011). Interventions for preventing obesity in children (review). Cochrane collaboration.

[29] Brownson RC, Seiler R, Eyler AA (2010). Measuring the impact of public health policy. Prev Chronic Dis.

[30] Alvaro C, Jackson LA, Kirk S, McHugh TL, Hughes J, Chircop A, Lyons RF (2011). Moving Canadian governmental policies beyond a focus on individual lifestyle: some insights from complexity and critical theories. Health Promot Int.

[31] Booth SL, Sallis JF, Ritenbaugh C, Hill JO, Birch LL, Frank LD, Glanz K, Himmelgreen DA, Mudd M, Popkin BM, Rickard KA, St Jeor S, Hays NP (2001). Environmental and societal factors affect food choice and physical activity: rationale, influences, and leverage points. Nutr Rev.

[32] Kamath CC, Vickers KS, Ehrlich A, McGovern L, Johnson J, Singhal V, Paulo R, Hettinger A, Erwin PJ, Montori VM (2008). Behavioral interventions to prevent childhood obesity. A systematic review and meta-analyses of randomized trials. J Clin Endocrinol Metab.

[33] McGovern L, Johnson JN, Paulo R, Hettinger A, Singhal V, Kamath C, Erwin PJ, Montori VM (2008). Treatment of pediatric obesity: a systematic review and meta-analysis of randomized trials. J Clin Endocrinol Metab.

[34] Witham MD, Avenell A (2010). Interventions to achieve long-term weight loss in obese older people: a systematic review and meta-analysis. Age and Ageing.

[35] Wu T, Gao X, Chen M, van Dam RM (2009). Long-term effectiveness of diet-plus-exercise interventions vs. diet-only interventions for weight loss: a meta-analysis. Obes Rev.

[36] Epstein LH, Leddy JJ, Temple JL, Faith MS (2007). Food reinforcement and eating: a multilevel analysis. Psychological Bulletin.

[37] Epstein LH, McKenzie SJ, Valoski A, Klein KR, Wing RR (1994). Effects of mastery criteria and contingent reinforcement for family-based child weight control. Addictive Behaviors.

[38] Gonzalez A, Boyle MH, Georgiades K, Duncan L, Atkinson LR, MacMillan HL (2012). Childhood and family influences on body mass index in early adulthood: findings from the Ontario Child Health Study. BMC Public Health.

[39] Swinburn BA, Sacks G, Hall KD, McPherson K, Finegood DT, Moodie ML, Gortmaker SL (2011). The global obesity pandemic: shaped by global drivers and local environments. Lancet.

[40] Bronfenbrenner U (1977). Toward an experimental ecology of human development. Am Psychol.

[41] Huang T, Story MT (2010). A journey just started: renewing efforts to address childhood obesity. Obesity.

[42] Nathan SA, Develin E, Grove N, Zwi AB (2005). An Australian childhood obesity summit: the role of data and evidence in ‘public’ policy making. Aust New Zealand Health Policy.

[43] Economos CD, Hyatt RR, Goldberg JP, Must A, Naumova EN, Collins JJ, Nelson ME (2007). A community intervention reduces bmi z-score in children: Shape Up Somerville first year results. Obesity.

[44] Economos CD, Hyatt RR, Must A, Goldberg JP, Kuder J, Naumova EN, Collins JJ, Nelson ME (2013). Shape Up Somerville two-year results: a community-based environmental change intervention sustains weight reduction in children. Prev Med.

[45] Sanigorski AM, Bell AC, Kremer PJ, Cuttler R, Swinburn BA (2008). Reducing unhealthy weight gain in children through community capacity-building: results of a quasi-experimental intervention program, Be Active Eat Well. Int J Obes.

[46] Borys JM, Le Bodo Y, Jebb SA, Seidell JC, Summerbell C, Richard D, De Henauw S, Moreno LA, Romon M, Visscher TLS, Raffin S, Swinburn B, the EEN Study Group (2012). EPODE approach for childhood obesity prevention: methods, progress and international development. Obes Rev.

[47] Haby MM, Doherty R, Welch N, Mason V (2012). Community-based interventions for obesity prevention: lessons learned by Australian policymakers. BMC Research Notes.

[48] Nutbeam D (1998). Evaluating health promotion: progress, problems and solutions. Health Promot Int.

[49] Van Koperen TM, Jebb SA, Summerbell CD, Visscher TLS, Romon M, Borys JM, Seidell JC (2013). Characterizing the EPODE logic model: unravelling the past and informing the future. Obes Rev.

[50] Gracia-Marco L, Mayer J, Vicente-Rodriguez G, Vinck J, Pettigrew S, Renes RJ, Le Bodo Y, Moreno LA, Borys JM, Le Bodo Y, De Henauw S, Moreno LA, Romon M, Seidell JC, Visscher TLS (2011). Methods and Social Marketing. Preventing Childhood Obesity: EPODE European Network Recommendations.

[51] Glaser B, Strauss A (1967). The Discovery of Grounded Theory.

[52] Lincoln Y, Guba E (1985). Naturalistic Inquiry.

[53] Glanz K, Bishop DB (2010). The Role of Behavioral Science Theory in Development and Implementation of Public Health Interventions. Annu Rev Public Health.

[54] Jacobs JA, Jones E, Gabella BA, Spring B, Brownson RC (2012). Tools for implementing an evidence-based approach in public health practice. Prev Chronic Dis.

[55] Yamey G (2012). What are the barriers to scaling up health interventions in low and middle income countries? A qualitative study of academic leaders in implementation science. Global Health.

[56] Brownson RC, Fielding JE, Maylahn CM (2009). Evidence-based public health: a fundamental concept for public health practice. Annu Rev Public Health.

[57] Lefebvre RC, Flora JA (1988). Social marketing and public health intervention. Health Educ Behav.

[58] Moodie R, Stuckler D, Monteiro C, Sheron N, Neal B, Thamarangsi T, Lincoln P, Casswell S, on behalf of The Lancet NCD Action Group (2013). Profits and pandemics: prevention of harmful effects of tobacco, alcohol, and ultra-processed food and drink industries. Lancet.

[CR59] The pre-publication history for this paper can be accessed here:http://www.biomedcentral.com/1471-2458/14/757/prepub

